# Projection of the year 2050 burden of diabetes in the US adult population: dynamic modeling of incidence, mortality, and prediabetes prevalence

**DOI:** 10.1186/1478-7954-8-29

**Published:** 2010-10-22

**Authors:** James P Boyle, Theodore J Thompson, Edward W Gregg, Lawrence E Barker, David F Williamson

**Affiliations:** 1 Division of Diabetes Translation, National Center for Chronic Disease Prevention and Health Promotion, Centers for Disease Control and Prevention, Mailstop K10, 4770 Buford Highway NE, Atlanta GA 30341 USA; 2 Hubert Department of Global Health, Rollins School of Public Health RM 740, Emory University, Atlanta GA 30329 USA

## Abstract

**Background:**

People with diabetes can suffer from diverse complications that seriously erode quality of life. Diabetes, costing the United States more than $174 billion per year in 2007, is expected to take an increasingly large financial toll in subsequent years. Accurate projections of diabetes burden are essential to policymakers planning for future health care needs and costs.

**Methods:**

Using data on prediabetes and diabetes prevalence in the United States, forecasted incidence, and current US Census projections of mortality and migration, the authors constructed a series of dynamic models employing systems of difference equations to project the future burden of diabetes among US adults. A three-state model partitions the US population into no diabetes, undiagnosed diabetes, and diagnosed diabetes. A four-state model divides the state of "no diabetes" into high-risk (prediabetes) and low-risk (normal glucose) states. A five-state model incorporates an intervention designed to prevent or delay diabetes in adults at high risk.

**Results:**

The authors project that annual diagnosed diabetes incidence (new cases) will increase from about 8 cases per 1,000 in 2008 to about 15 in 2050. Assuming low incidence and relatively high diabetes mortality, total diabetes prevalence (diagnosed and undiagnosed cases) is projected to increase from 14% in 2010 to 21% of the US adult population by 2050. However, if recent increases in diabetes incidence continue and diabetes mortality is relatively low, prevalence will increase to 33% by 2050. A middle-ground scenario projects a prevalence of 25% to 28% by 2050. Intervention can reduce, but not eliminate, increases in diabetes prevalence.

**Conclusions:**

These projected increases are largely attributable to the aging of the US population, increasing numbers of members of higher-risk minority groups in the population, and people with diabetes living longer. Effective strategies will need to be undertaken to moderate the impact of these factors on national diabetes burden. Our analysis suggests that widespread implementation of reasonably effective preventive interventions focused on high-risk subgroups of the population can considerably reduce, but not eliminate, future increases in diabetes prevalence.

## Background

People with diabetes often develop diverse microvascular, macrovascular, and neuropathic complications that seriously erode quality of life. The high prevalence, high incidence, chronicity, and long-term implications for health and health care costs make diabetes a major concern for the United States and much of the developed and developing world [1,2]. In 2007, diabetes cost the US in excess of $174 billion [3]. Diabetes is expected to take an increasingly large financial toll in the future, particularly on older adults in developed countries and on working-age adults in developing countries [4]. Accurate projections of diabetes burden are essential to policymakers planning for future health care needs and costs.

Several future projections of the prevalence, incidence, and total number of diabetes cases for the US and other countries have been carried out [5-8]. However, previous estimates for the US are likely to be outdated because they relied on 1990 census projections. These older census projections overestimate current mortality rates and do not account for the increasing size of the Hispanic and foreign-born US populations at higher risk for developing diabetes. Previous estimates assumed no increase in diabetes incidence and did not consider the impact of undiagnosed diabetes on total diabetes prevalence. In addition, earlier estimates ignored the substantial variation in diabetes incidence occurring between the subpopulation with normal glucose levels and the subpopulation with prediabetes.

To overcome these limitations and provide contemporary, realistic estimates of the growth of the national diabetes burden, we constructed a system of dynamic equations that incorporate initial prevalence (percentage of population with diabetes, both diagnosed and undiagnosed), incidence (percentage of population with newly diagnosed diabetes), migration, mortality, and prevalence of prediabetes. These equations model the future burden of diabetes on US adults through 2050. We also consider the effect of a hypothetical, large-scale preventive intervention.

## Materials and methods

### Data sources

The data sources for this study include the US Census Bureau [9] and the Centers for Disease Control and Prevention (CDC) [10]. Census data are based on the 2000 census and include estimates of the 2007 population and estimates of mortality rates, net migration, and births from 2008 through 2050. CDC data include estimates and standard errors of incidence rates of diagnosed diabetes for the US adult population (aged 18-79 years) from 1980 through 2007. The application of these data depended on a transition matrix that was based on a literature review. This matrix contains estimates of the rates of transition from having no diabetes, prediabetes, and undiagnosed diabetes to having diagnosed diabetes, as well as the risk of mortality associated with different glycemic and diabetic states.

### Incidence projections

Let the annual incidence rate of diagnosed diabetes and its estimated standard error be denoted by (*y_t_, s_t_*) for *t *= 1,...,28, corresponding to years 1980 through 2007. We fit a logistic growth curve with asymptote *ρ *using Bayesian methods [11]. The logistic growth curve restricts incidence rates to be monotonic over time. An informative prior distribution, putting approximately 95% of its mass between the 2007 US rate ( 0.0078) and the Pima Indian rate (0.025) [12] was used for *ρ*. The Pima Indian incidence rate was used because the Pima have the highest diabetes incidence among subpopulations of the US. A normal distribution was assumed for *y*_*t *_Incidence projections are denoted as *μ*_*t *_for *t *= 29,...,71, corresponding to years 2008 through 2050. This model is described by:

Diffuse normal prior distributions were used for *λ*_0 _and *λ*_1_. Four different specifications for *ε*_*t *_were considered: unstructured, first order autoregressive (AR(1)), first order moving average (MA(1)), and first order autoregressive-moving average (ARMA(1,1)). Graphical displays of the posterior distributions of the residuals, *y*_*t *_- *μ*_*t*_, and the residual sum of squares were used to compare models. The AR(1) and ARMA(1,1) were indistinguishable and clearly fit better than the unstructured and MA(1) models. We used the AR(1) model because it contains fewer parameters than the ARMA(1,1) model. The AR(1) model's residuals did not exhibit serial correlation and were all less than 0.0005 in absolute value, indicating good model fit. Posterior distributions of the projected incidences of diagnosed diabetes are simulated as part of the model fitting process. Modeling was done using WinBUGS software [13].

### Diabetes projections

We constructed a series of dynamic models that consisted of systems of difference equations in time that are similar to models described elsewhere [14,15] to project the future burden of diabetes in the United States. These models incorporated initial US prevalence, modeled incidence projections, census-based mortality projections, and a time-varying transition matrix that differentially allotted the population into states of normal glucose tolerance, prediabetes, and undiagnosed diabetes/diagnosed diabetes. A three-state model partitioned the US adult population into the states of no diabetes, undiagnosed diabetes, and diagnosed diabetes so that the projected total adult population would agree with census projections. A four-state model extended the three-state model by splitting the adult population with no diabetes into high- and low-risk groups. Finally, we present preliminary results from a five-state model that represents the potential effect of a hypothetical preventive intervention delivered to all people with impaired fasting glucose (IFG), a group at high risk for future development of diabetes. If half of the people with IFG participated in an intervention and their incidence was reduced by 50%, it would be roughly equivalent to a 25% reduction in all people with IFG. Therefore, we assumed that the hypothetical intervention would reduce by 25% the annual incidence of diabetes in people with IFG.

Key assumptions for the models follow. First, people cannot move from diabetes to nondiabetes; this assumption is reasonable because remission is extremely rare. Second, the relative risks of death for the two diabetes states versus the no diabetes state are constant over time. The number of ways that relative risk might vary over time is infinite. In the absence of data about which of these patterns of varying relative risk to choose, we chose the simplest one: no time variation. Third, the transition rates to diagnosed or undiagnosed diabetes for nondiabetics are constant multiples of the transition rate to diagnosed diabetes for undiagnosed diabetics. This assumption implies the proportion of diagnosed diabetics among all new diabetics in any given year is constant over time.

A detailed description of these models, including all assumptions, references for key parameter estimates, and algebraic derivations, are presented in Appendix 1 and Appendix 2. The programs for implementing the models were written in GAUSS [16].

## Results

**Figure 1:**
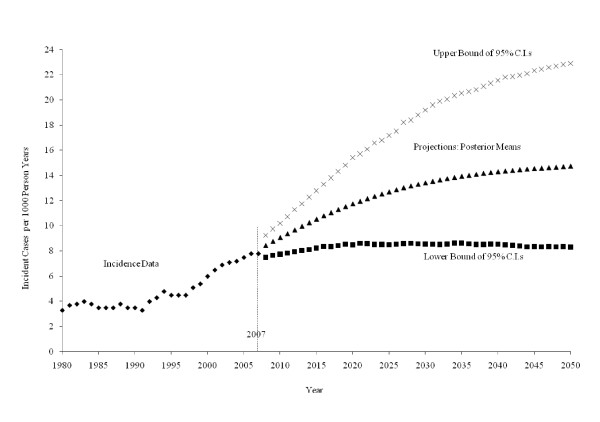
**Incident cases of diagnosed diabetes per 1,000 people, 1980-2007, and three scenarios for projected cases per 1,000, 2008 -2050: a middle scenario (posterior means) and low and high scenarios (lower and upper limits of 95% Bayesian confidence intervals) from the projection model of diagnosed diabetes incidence**.

Figure 1 plots the historical incident cases of diagnosed diabetes per 1,000 people for 1980 through 2007. It also plots three projection scenarios for 2008 through 2050: *low incidence*, which is the 2.5^th ^percentile of the posterior distribution; *middle incidence*, which is the posterior mean; and *high incidence*, which is the 97.5^th ^percentile of the posterior distribution. Historical incidence rates range from 3.3 cases per 1,000 in 1980 to 7.8 cases per 1,000 in 2007. The middle incidence scenario increases steadily over the projection horizon, from 8.4 cases per 1,000 in 2008 to 14.7 cases per 1,000 in 2050. The low incidence scenario remains relatively flat, with an average incidence of 8.4 cases per 1,000, while the high incidence scenario projects extreme increases in incidence from 9.2 to 22.9 cases per 1,000 for the years 2008 through 2050.

We denote the relative risk of death for individuals with undiagnosed diabetes versus those without diabetes as *r*_*1 *_and the relative risk of death for individuals with diagnosed diabetes versus those without diabetes as *r*_*2*_. Published results [17] lead to estimates *r*_1 _= 1.77 and *r*_2 _= 2.11, and we refer to this set of values as *low mortality risk*. We also set *r*_1 _= 1.00 and *r*_2 _= 4.08, consistent with projections from Narayan et al [7] aggregated to the US adult population aged 18-79 years, and we refer to this set of values as *high mortality risk *(see Appendix 1 for more details).

**Figure 2:**
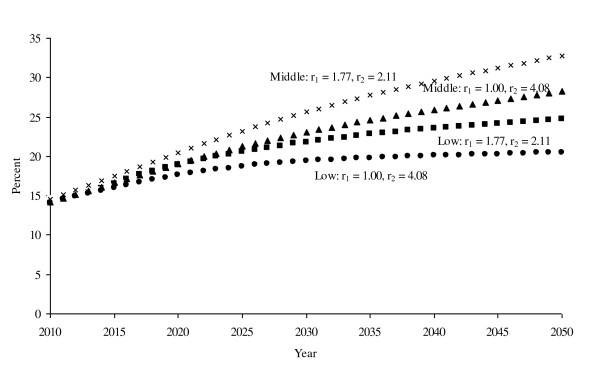
**Projections of total diabetes prevalence as a percentage of the total US adult population for four scenarios: low incidence projections and r_1 _= 1.77, r_2 _= 2.11; low incidence projections and r_1 _= 1.00, r_2 _= 4.08; middle incidence projections and r_1 _= 1.77, r_2 _= 2.11; middle incidence projections and r_1 _= 1.00, r_2 _= 4.08**.

Table 1 provides projections of the numbers (in millions) of people with no diabetes, undiagnosed diabetes, or diagnosed diabetes every fifth year from 2010 through 2050. We provide projections for the low and middle incidence scenarios, both for low mortality risk and high mortality risk, for four scenarios. Results for the high incidence projections are reported in this section only. All four model scenarios indicate at least a doubling, and in some cases an even greater increase, in the number of people with diagnosed diabetes from 2010 through 2050. When compared to this rapid growth, the projected number of people with undiagnosed diabetes grows slowly under all four scenarios. In Figure 2, the prevalence of any diabetes (diagnosed or undiagnosed) increases from 14.1%-14.5% in 2010 to 24.7%, 20.5%, 32.8%, and 28.3% in 2050 under, respectively, the low incidence scenarios (low and high mortality risk) and middle incidence scenarios (low and high mortality risk).

**Table 1:** Projections from the Three-State Model of Numbers of People in Millions with No Diabetes, Undiagnosed Diabetes, and Diagnosed Diabetes for Selected Years

Year	Relative Risk r_1_	Relative Risk **r_2_**	No Diabetes (Low, Middle)	Undiagnosed Diabetes (Low, Middle)	Diagnosed Diabetes (Low, Middle)	Total US Adult Population
2010	1.77	2.11	(191.4, 191.2)	(12.0, 11.5)	(20.3, 21.0)	223.7
	1.00	4.08	(192.1, 191.9)	(12.1, 11.6)	(19.5, 20.2)	
2015	1.77	2.11	(196.1, 194.6)	(13.1, 12.2)	(26.6, 29.1)	235.9
	1.00	4.08	(198.1, 196.6)	(13.3, 12.4)	(24.4, 26.8)	
2020	1.77	2.11	(200.7, 196.9)	(13.9, 12.7)	(32.9, 37.9)	247.5
	1.00	4.08	(204.0, 200.3)	(14.3, 13.0)	(29.2, 34.1)	
2025	1.77	2.11	(205.4, 198.5)	(14.4, 13.0)	(38.7, 47.0)	258.5
	1.00	4.08	(210.2, 203.6)	(14.9, 13.5)	(33.4, 41.4)	
2030	1.77	2.11	(209.5, 199.3)	(14.7, 13.1)	(43.7, 55.5)	267.9
	1.00	4.08	(216.0, 206.2)	(15.4, 13.7)	(36.5, 48.0)	
2035	1.77	2.11	(213.9, 200.1)	(15.0, 13.2)	(48.1, 63.6)	276.9
	1.00	4.08	(222.1, 208.9)	(15.8, 14.0)	(39.1, 54.1)	
2040	1.77	2.11	(218.3, 201.0)	(15.2, 13.3)	(52.0, 71.2)	285.5
	1.00	4.08	(228.2, 211.6)	(16.2, 14.2)	(41.1, 59.7)	
2045	1.77	2.11	(223.6, 202.7)	(15.5, 13.4)	(55.6, 78.6)	292.9
	1.00	4.08	(235.1, 215.0)	(16.6, 14.4)	(43.1, 65.4)	
2050	1.77	2.11	(230.6, 206.0)	(16.0, 13.7)	(59.7, 86.6)	306.3
	1.00	4.08	(243.5, 219.7)	(17.2, 14.8)	(45.6, 71.8)	

Note: There are four scenarios included (1) low incidence projections and r_1 _= 1.77, r_2 _= 2.11, (2) low incidence projections and r_1 _= 1.00, r_2 _= 4.08, (3) middle incidence projections and r_1 _= 1.77, r_2 _= 2.11, (4) middle incidence projections and r_1 _= 1.00, r_2 _= 4.08. Entries in the last column are the Census projections of the total US adult population.

Table 2 (all results in thousands) displays results from the hypothetical preventive intervention assessing the impact of intervention on diabetes incidence. The fourth column displays the number of incident cases of diabetes under the low and middle incidence scenarios with low and high mortality risk. The fifth column does the same for the number of incident cases and assumes that our hypothetical intervention is in place. The sixth column displays the difference between the fifth and sixth columns, which is the number of incident cases the intervention prevented or delayed. For example, using the projected middle incidence scenario and low mortality risk, 3,490,900 incident cases would be reported in 2050 with no intervention and 3,146,100 incident cases would be reported with intervention, for a net reduction of 344,800 incident cases of diabetes.

**Table 2:** Projections for Selected Years of Incident Cases in Thousands from the Adult Population with No Diabetes from the No-Intervention Model (Three-State Model) and the Preventive Intervention Model (Five-State Model)

Year	Relative	Relative	No-Intervention	Intervention	Difference (Low, Middle)
	Risk r_1_	Risk r_2_	Incident Cases (Low, Middle)	Incident Cases (Low, Middle)	
2010	1.77	2.11	(2018.4, 2145.7)	(1681.6, 1787.9)	(336.8, 357.8)
	1.00	4.08	(2021.1, 2148.4)	(1683.8, 1790.1)	(337.3, 358.3)
2015	1.77	2.11	(2095.4, 2468.1)	(1773.2, 2093.2)	(322.2, 374.9)
	1.00	4.08	(2106.8, 2481.9)	(1782.7, 2104.9)	(324.1, 377.0)
2020	1.77	2.11	(2143.3, 2721.9)	(1833.3, 2341.0)	(310.0, 380.9)
	1.00	4.08	(2164.9, 2752.2)	(1851.7, 2366.9)	(313.2, 385.3)
2025	1.77	2.11	(2176.1, 2933.5)	(1875.8, 2551.9)	(300.3, 381.6)
	1.00	4.08	(2208.9, 2984.9)	(1904.1, 2596.6)	(304.8, 388.3)
2030	1.77	2.11	(2230.0, 3098.4)	(1933.6, 2721.5)	(296.4, 376.9)
	1.00	4.08	(2276.0, 3175.1)	(1973.5, 2789.1)	(302.5, 386.0)
2035	1.77	2.11	(2300.8, 3225.0)	(2004.4, 2855.9)	(296.4, 369.1)
	1.00	4.08	(2361.4, 3329.7)	(2057.3, 2949.2)	(304.1, 380.5)
2040	1.77	2.11	(2334.3, 3323.2)	(2041.4, 2963.2)	(292.9, 360.0)
	1.00	4.08	(2408.8, 3456.9)	(2107.0, 3083.6)	(301.8, 373.3)
2045	1.77	2.11	(2341.5, 3401.4)	(2054.0, 3050.5)	(287.5, 350.9)
	1.00	4.08	(2428.3, 3562.9)	(2130.6, 3197.3)	(297.7, 365.6)
2050	1.77	2.11	(2403.8, 3490.9)	(2113.8, 3146.1)	(290.0, 344.8)
	1.00	4.08	(2502.6, 3677.2)	(2201.7, 3316.8)	(302.9, 360.4)

Note: There are four scenarios included (1) low incidence projections and r_1 _= 1.77, r_2 _= 2.11, (2) low incidence projections and r_1 _= 1.00, r_2 _= 4.08, (3) middle incidence projections and r_1 _= 1.77, r_2 _= 2.11, (4) middle incidence projections and r_1 _= 1.00, r_2 _= 4.08.

## Discussion

Our estimates of diabetes prevalence paint a sobering picture of the future growth of diabetes. Under an assumption of low incidence and relatively high diabetes mortality, total prevalence is projected to increase to 21% of the US adult population by 2050. On the other hand, if recent increases in diabetes incidence continue (middle incidence projections) and diabetes mortality ratios are relatively low, diabetes prevalence will increase to 33% by 2050. The middle-ground (low incidence with low mortality or middle incidence with high mortality) scenarios project a prevalence of 25% to 28% by 2050. In each of the scenarios, the increases are, in part, attributable to demographic changes. The population of the United States is aging, and older adults are more likely to develop diabetes than younger adults. The size of minority populations in the United States also is growing, and some minorities are at greater risk of developing diabetes than non-Hispanic whites. Finally, mortality among people with diabetes is declining. The result is that people with diabetes live longer and contribute to prevalence for longer periods of time.

Two previous diabetes forecasts have linearly extrapolated historical prevalence trends. In 2004, Wild et al [18] projected a 114% increase in the number of people with diabetes from 2000 through 2030 worldwide. More recent estimates on behalf of the International Diabetes Federation suggested that, from 2010 through 2030, increases in diabetes prevalence will range from a 20% increase in Europe to an almost doubling of prevalence in Africa and the Middle East [4]. Others have built dynamic models incorporating incidence, mortality, and migration. A discrete, three-state Markov model that stratified by age, sex, and race/ethnicity projected an approximate doubling in US prevalence by 2050 [6,7]. In 2007, a model using NHANES III as a starting point and a midstream validation using NHANES 1999-2002 data projected an approximate 50% increase in the next 20 years in the United States [19]. Magliano et al [20] projected diabetes prevalence in Australia increasing from 10.1% in 2010 to 17.0% in 2025, a trend similar to our low incidence-high mortality risk results.

Our models, which include the ability to evaluate preventive interventions, suggest that the future prevalence of diagnosed diabetes could be significantly worse than previously suggested. A large increase in diabetes prevalence could be driven by multiple factors, including increasing incidence, better detection, and in-migration. Our updated model includes a higher level of incidence based on the CDC National Diabetes Surveillance System and projects lower future mortality rates than were used in previous models based on US Census data. In addition, our model assumes that the mortality rate of the diabetic population will decline at least as much as that of the nondiabetic population (i.e., the mortality rate ratio associated with diabetes will be constant). Recent comparison of US cohorts suggests that this assumption is reasonable [21].

The projected loss in quality of life and the projected costs of providing health care could be significant. Increased efforts in primary prevention of diabetes can help to decrease loss in quality of life and the future cost of providing care for people with diabetes. Indeed, such efforts are essential if we hope to moderate or slow the growth of diabetes prevalence. However, as Table 2 indicates, prevention efforts can be reasonably expected to moderate, but not prevent, future growth in the number of people with the disease.

Our five-state model made the assumption that a hypothetical intervention would reach 100% of those with IFG and would reduce the annual incidence of diabetes in this group by 25%. Future efforts to refine our modeling approach will focus on more realistic specification of intervention scenarios applied to a variety of population subgroups at high risk of developing diabetes. Had we split the population at high risk into intervention and nonintervention subsets, we would have obtained estimates between the no intervention and intervention cases in Table 2. Thus, column six of Table 2 can be viewed as an upper bound on the number of incident diabetes cases that a hypothetical intervention could prevent.

Our model is subject to several limitations. Cases of diabetes in people younger than age 18 or older than age 79 years were not considered. Although diabetes in the young is rare, and a relatively small portion of the US population is aged 80 years or older, these numbers might not be negligible. Our model made many reasonable but untestable assumptions. For example, we assumed that the relative risks of death for those with detected or undetected diabetes, compared to those without diabetes, are constant over time. We assumed that the observed increase in diabetes incidence fits a logistic growth curve. Given the logistic model, we could have chosen either a more or less precise prior for *ρ*. While assumptions other than the ones we made could have been made, we are aware of no data sources that would support such assumptions. We assumed that census estimates for the intercensal years between 2000 and 2009 and the census projections of net migration, births, and death rates for 2010 and beyond are accurate. We also assumed no feedback effect; for example, the increasing prevalence of diabetes could conceivably contribute to greater awareness of diabetes, which could, in turn, reduce the incidence rate or could result in fewer cases of diabetes remaining undiagnosed. Finally, our model implicitly assumes that the future will resemble the past. Changes in the levels of circulating glucose or A1c considered to define diabetes could change the prevalence of both diagnosed and undiagnosed diabetes. Major upheavals, such as an epidemic or natural disaster that substantially changed birth or death rates or a dramatic social change that invalidated census projections or caused other changes in the way people lead their lives, could have correspondingly major impacts on the outcomes of our model.

We performed a sensitivity analysis that assumed 98% prior probability for *ρ*, the asymptote of the incidence, in the interval (0.0078, 0.025). The sensitivity analysis produced no practical difference in the incidence projections. We also investigated the sensitivity of our model by considering low mortality risk, high mortality risk, middle incidence projections, and low incidence projections. The additional model assumptions are justified in the appendices. A formal sensitivity analysis of all assumptions would present a substantial technical challenge. We believe it is more useful to policymakers for us to present the results of our four model scenarios.

We anticipate that the modeling methods described here could be used by other countries, especially those with reliable census estimates, to estimate future diabetes burden, as well as the potential effects of interventions to reduce disease burden. Country-specific data elements could be easily substituted for the data elements we used to develop a model that fit US population dynamics. Further, a modified form of this model might be applicable to other chronic, near-irreversible, and sometimes undiagnosed conditions such as heart disease.

## Conclusion

We project that, over the next 40 years, the prevalence of total diabetes (diagnosed and undiagnosed) in the United States will increase from its current level of about 1 in 10 adults to between 1 in 5 and 1 in 3 adults in 2050. The health care costs of a person with diagnosed diabetes are approximately 2.3 times that of a person without [3]. Although a formal projection of costs is beyond the scope of this analysis, the total societal cost of diabetes is likely to dramatically increase over the coming decades. The increases in diabetes prevalence projected here are largely attributable to a combination of three key demographic factors, including aging of the US population, increasing size of higher-risk minority populations, and declining mortality among people with diabetes. Although these demographic factors reflect underlying improvements in the health of the US population, effective strategies will need to be undertaken to moderate the impact of these factors on national diabetes burden. Our analysis suggests that widespread implementation of reasonably effective preventive interventions focused on high-risk subgroups of the population may not eliminate, but might considerably reduce, future increases in diabetes prevalence.

## Competing interests

The authors declare that they have no competing interests.

## Authors' contributions

JPB developed and programmed the multistate dynamic models, participated in study design and coordination, and helped draft the manuscript. TJT developed and programmed the incidence projection model, participated in study design and coordination, and helped draft the manuscript. EWG participated in study design and coordination and helped draft the manuscript. LEB participated in study design and coordination and helped draft the manuscript. DFW conceived of the study, participated in study design and coordination, and helped draft the manuscript. All authors contributed to critical revision of the draft manuscript, and all authors read and approved the final manuscript.

## Appendix 1

### Three-state model

The US adult population is modeled at 1-year intervals starting at year *t *= 28 (2007) and ending at *t *= 71 (2050). Specifically, define the following numbers of people in various states, rates, and flows. All rates are annual and flows occur during year *t*, i.e., in the interval (*t*-1,*t*].

*X*(*t*) = the number of adults without diabetes at time = *t*.

*Z*(*t*) = the number of adults with undiagnosed diabetes at time = *t*.

*Y*(*t*) = the number of adults with diagnosed diabetes at time = *t*.

*b*(*t*) = Census projection of the number of adults turning 18 (births) during year *t*.

*m*(*t*) = Census projection of the number of adults migrating into the United States during year *t*.

*d*(*t*) = Census projected death rate for the US resident population aged 18-79 years.

*γ*(*t*) = nondiabetes death rate among *X*(*t*-1).

*r*_*1*_*γ*(*t*) = diabetes death rate among *Z*(*t*-1); *r*_*2*_*γ*(*t*) = diabetes death rate among *Y*(*t*-1); *r*_*1 *_and *r*_*2 *_are relative risks.

*i*(*t*) = incidence rate of diagnosed diabetes among *X*(*t*-1) or *Z*(*t-*1), denoted earlier by .

*I*(*t*)= incidence rate of all diabetes (undiagnosed or diagnosed) among *X*(*t*-1).

*f*_*x*_(*t*) = proportion of *b*(*t*) without diabetes.

*f*_*z*_(*t*) = proportion of *b*(*t*) with undiagnosed diabetes.

*f*_*y*_(*t*) = proportion of *b*(*t*) with diagnosed diabetes.

*g*_*x*_(*t*) = proportion of *m*(*t*) without diabetes.

*g*_*z*_(*t*) = proportion of *m*(*t*) with undiagnosed diabetes.

*g*_*y*_(*t*) = proportion of *m*(*t*) with diagnosed diabetes.

The relations *f*_*x*_(*t*)+ *f*_*z*_(*t*)+ *f*_*y*_(*t*) = 1 and *g*_*x*_(*t*)+ *g*_*z*_(*t*)+ *g*_*y*_(*t*) = 1 ensure consistency with the census projections. Define , the prevalence of diabetes at time *t*; , the prevalence of undiagnosed diabetes at time *t*; , the prevalence of diagnosed diabetes at time *t*. Clearly θ(*t*) = θ_1_(*t*) + θ_2_(*t*).

Consider the following transition matrix:.

Note that this matrix displays the distribution of the beginning year stocks (rows) to the ending year stocks (the columns), and thus, the transition rates in each row must be nonnegative and add to unity for each year *t*. Some assumptions about transition rates are apparent. First, people cannot move from diabetes to nondiabetes; this assumption is reasonable because remission is extremely rare. Second, the relative risks of death for the two diabetes states are constant over time. No data were found to support time varying relative risks, but published results [17] lead to estimates *r*_1 _= 1.77 and *r*_2 _= 2.11. We also set *r*_1 _= 1.00 and *r*_2 _= 4.08 in a sensitivity analysis, consistent with projections from Narayan et al [6] aggregated to the US adult population aged 18-79 years. Third, the transition rates to diagnosed or undiagnosed diabetes for nondiabetics are constant multiples of the transition rate to diagnosed diabetes for undiagnosed diabetics. General time varying rates for these transitions were not available, but, as detailed below, estimates of ξ_1 _and ξ_2 _could be obtained. This assumption implies the proportion of diagnosed diabetics among all new diabetics in any given year *t *is constant over time, i.e.,  or . To calculate η, assume that 95% of the time a person spends less than seven years in the undiagnosed diabetes state [22] and that the hazard rate for moving from undiagnosed to diagnosed diabetes is constant over time. This equates to a 0.19 probability of moving to the diabetes state within six months. The transition matrix leads to the system of first order difference equations

with initial conditions *X*(28), *Y*(28), *Z*(28).

Consistency with census projections of the number of US adults *N*(*t*) requires *X*(*t*) *+ Z*(*t*) *+ Y*(*t*) *= N*(*t*) where

This is guaranteed if the following two equations are satisfied:

But the second equation is equivalent to

(with a little algebra). The first is a consequence of

where the values .398 and .129 come from [23]. These equations imply

with *N*(28) = 215,750,418, the census estimate of the 2007 US adult population.

Now, given a projection *i*(*t*), then

and *β*(*t*) is

Noting that, in general,

then

where the value .0106 is derived in Appendix 2. Solving this equation for x_1 _yields.

Thus, *ξ*_1_, *ξ*_2 _are determined.

Finally, the distributions of births and net migration across the three subpopulations for each year are determined by *f_x_(t) *= 1, *f_z_(t) *= 0, *f_y_(t) *= 0

and 

The first set of equations reflects our baseline assumption that all incoming births are nondiabetic. The second set of equations simply distributes the net migration for year *t *according to the proportions in each state at the beginning of the year. All parameters in the model are thus determined and the system of equations described earlier can be used to calculate the model projections.

### Four-state model

The four-state model expands the three-state model by splitting *X*(*t*) = *HX*(*t*) + *LX*(*t*), where *HX(t) = p(t)X(t) *is the number of adults in a high-risk group (e.g., those with IFG) and *LX(t) = *[1-*p(t)*] *X(t) *is the number in a low-risk group (e.g., those without IFG). This must be done so that *X*(*t*), *Z*(*t*), and *Y*(*t*) are as in the three-state model.

To this end, consider the transition matrix

Note that the death rates are equal to those in the three-state model when

Also, transition rates to diabetes are the same as in the three-state model if the following relation holds:

One additional assumption is made to determine *h*(*t*) and *l*(*t*), that the ratio of the incidence rates to any diabetes of the high-risk group to the low-risk group is constant over time (constant relative incidence). Specifically,

The two equations above yield the expressions

The constant *c *can be calculated as follows. Let λ equal the 2008 incidence rate from the high-risk group to any diabetes and *p*(28) the proportion of the nondiabetic population at high risk in 2007. For example, for IFG λ = .0287 and *p*(28) = .257 from Appendix 2. Then, (ξ_1 _+ ξ_2_)*h*(29)*p*(28)*X*(28) = λ*p*(28)*X*(28) implies . But  and .

For the risk strata IFG, this gives *c *= 6.6. All of this ensures that the relevant transition rates from the nondiabetic population to the diabetic population are the same as in the three-state model. Since death rates for these populations are as in the three-state model, the four-state model properly expands the three-state model when

and

To complete the model, the α's must be chosen. The constraints are

Let

Then there exists *q(t) *in the interval (0,1) and *s(t) *in (0,1) such that

For the high-risk population with IFG, we set *q*(*t*) = *q *= .93 to get α_1_(29) = .89 from [23]. Finally, assuming the proportion *p*(*t*) = .257 for all *t *≥ 28, it is easy to derive (details omitted)

The actual computation of the four-state model can be implemented through the system of difference equations

with initial conditions *HX*(28) *= p*(*28*) *X*(28), *LX*(28)=[1-*p*(28)] *X*(28), *Y*(28), *Z*(28).

### Five state model (preventive intervention)

Given the outputs from the two previous baseline models, consider the following transition matrix reflecting intervention on the high-risk population. Note that *IX*(*t*) now denotes the number of people in the high-risk or intervention group. Also, a new state has been added with *LXI*(*t*) equals the number of people in the intervention group who have regressed to low risk. The transition matrix is

where rows are labeled as columns with times *t*-1, and

State variables are relabeled because we expect the intervention model to deviate from the previous models. The initial conditions are the same as in the four-state model, with *LXI*(28) = 0. The associated system of difference equations is omitted and can be derived in the same way as in the previous two models.

## Appendix 2

There are no published nationally representative estimates for annual incidence of total diabetes (diagnosed or undiagnosed) for the US adult nondiabetic population or for subgroups defined by glycemic level. The one US estimate of diabetes incidence in 2007 for adults aged 18-79 (0.78%) applies only to diagnosed diabetes [10]. Therefore, we developed our own estimates of the annual incidence of total diabetes for the US nondiabetic adult population.

### Groups defined by glycemic level

Diabetes incidence in the entire population is a function of diabetes incidence in four mutually exclusive glycemic subgroups and their prevalence in the nondiabetic population. These subgroups are 1) *Normoglycemic *(NG): fasting blood glucose < 100 mg/dl and 2-hour post-challenge glucose < 140 mg/dl; 2) *Isolated Impaired Fasting Glucose *(IIFG): fasting blood glucose 100-125 mg/dl with 2-hour post-challenge blood glucose < 140 mg/dl; 3) *Isolated Impaired Glucose Tolerance *(IIGT): 2-hour post-challenge blood glucose 140-199 mg/dl with fasting blood glucose < 100 mg/dl; 4) *Combined IGT and IFG *(CIFGT): fasting blood glucose 100-125 mg/dl with 2-hour post-challenge blood glucose 140-199 mg/dl. We used 2005-2006 NHANES data, after excluding people with diagnosed and undiagnosed diabetes, to estimate glycemic group prevalences for the nondiabetic US adult population.

Gerstein et al [24] published estimates of the annualized relative risks of diabetes for IIFG, IIGT, and CIFGT with the NG subgroup as referent, but did not report the absolute annual incidence for the NG subgroup. To calculate the absolute incidence for each glycemic subgroup, the relative risk must be multiplied by the absolute incidence in the NG subgroup.

### Annual incidence in the normoglycemic

Santaguida et al [25] reported annualized incidence of diabetes from 42 studies in which the NG subgroup was included. We used 1999-2006 NHANES data to estimate that, among US adults aged ≥20 years, the mean age of the NG subgroup was 46 years. Four studies included by Santaguida et al reported a mean age of 43-48 years. One of these studies was on US Pima Indians, who are believed to have the world's highest diabetes incidence, and this study was excluded. We also identified one study published after Santaguida et al that reported a mean age of 50 years in the NG subgroup [20].

The annualized diabetes incidence for the NG subgroup from the four studies was 0.19%, 0.25%, 0.38%, and 0.64%. We used the median annual incidence of these four studies, 0.32%, to estimate diabetes incidence in the NG subgroup in the United States. Engberg et al [26] recently reported a nearly identical estimate of annual incidence of 0.3% for an NG subgroup from a population-based cohort in northern Europe with a mean age of 46 years.

### Annual incidence for dysglycemic subgroups

We multiplied the 0.32% annual diabetes incidence in the NG subgroup by the relative risks from Gerstein et al [24] of 5.5 (IIGT), 7.5 (IIFG), and 12.1 (CIFGT). The result was annual incidences of 1.76% (IIGT), 2.40% (IIFG), and 3.87% (CIFGT).

### Annual incidence in the total population

We multiplied the annual diabetes incidence of each independent glycemic subgroup (NG, IIGT, IIFG, and CIFGT) by its prevalence in the US nondiabetic population and summed the result, yielding an estimate of annual diabetes incidence of 1.06%. This finding is similar to that reported by recent population-based cohort studies. Bonora et al [27] studied adults aged 40-79 years in northeastern Italy and concluded that "...~1% of European white individuals aged 40-79 years develop Type 2 diabetes annually..." Maskarinec et al [28] studied a multi-ethnic sample of adults aged 45 -74 years in Hawaii and estimated an annual diabetes incidence of 1.04%.

## References

[B1] Engelgau MM Geiss LS Saaddine JB Boyle JP Benjamin JM Gregg EW Tierney EF Rios-Burrows N Mokdad AH Ford ES Imperatore G Narayan KMV The evolving diabetes burden in the United States Ann Intern Med 2004 140 945 950 15172919 10.7326/0003-4819-140-11-200406010-00035

[B2] Zimmet PZ Alberti KG Shaw JE Global and societal implications of the diabetes epidemic Nature 2001 414 782 787 10.1038/414782a 11742409

[B3] American Diabetes Association Economic costs of diabetes in the U.S. in 2007 Diabetes Care 2008 32 596 615 10.2337/dc08-9017 18308683

[B4] Shaw JE Sicree RA Zimmet PZ Global estimates of the prevalence of diabetes for 2010 and 2030 Diabetes Res Clin Pract 2009 87 4 14 10.1016/j.diabres.2009.10.007 19896746

[B5] Boyle JP Honeycutt AA Narayan KM Hoerger TJ Geiss LS Chen H Thompson TJ Projection of diabetes burden through 2050: impact of changing demography and disease prevalence in the U.S Diabetes Care 2001 24 11 1936 1940 10.2337/diacare.24.11.1936 11679460

[B6] Honeycutt AA Boyle JP Broglio KR Thompson TJ Hoerger TJ Geiss LS Narayan KMV A dynamic Markov model for forecasting diabetes prevalence in the United States through 2050 Health Care Management Science 2003 6 3 155 164 10.1023/A:1024467522972 12943151

[B7] Narayan KM Boyle JP Geiss LS Saaddine JB Thompson TJ Impact of recent increase in incidence on future diabetes burden: U.S., 2005-2050 Diabetes Care 2006 29 9 2114 2116 10.2337/dc06-1136 16936162

[B8] Magliano DJ Shaw JE Shortreed SM Nusselder WJ Liew D Barr ELM Zimmet PZ Peeters A Lifetime risk and projected population prevalence of diabetes Diabetologia 2008 51 12 2179 2186 10.1007/s00125-008-1150-5 18810385

[B9] U.S. Census Bureau. National Population Projections Released 2008 (Based on Census 2000) http://www.census.gov/population/www/projections/downloadablefiles.html

[B10] Centers for Disease Control and Prevention. Crude and Age-Adjusted Incidence of Diagnosed Diabetes per 1,000 Population Aged 18-79 Years, United States, 1980-2007 http://www.cdc.gov/diabetes/statistics/incidence/fig2.htm

[B11] Gelman A Carlin JB Stern HS Rubin DB Bayesian Data Analysis 2004 Boca Raton, FL: Chapman & Hall

[B12] Knowler WC Bennett PH Hamman RF Miller M Diabetes incidence and prevalence in Pima Indians: a 19-fold greater incidence than in Rochester, Minnesota Am J Epidemiol 1978 108 6 497 505 736028 10.1093/oxfordjournals.aje.a112648

[B13] Gilks WR Thomas A Spiegelhalter DJ A language and program for complex Bayesian modeling The Statistician 1994 43 1 169 178 10.2307/2348941

[B14] Bartholomew DJ Stochastic Models for Social Processes 1982 3 New York, NY: John Wiley & Sons

[B15] Sandefur JT Discrete Dynamical Modeling 1993 New York, NY: Oxford University Press

[B16] Aptech Systems Inc GAUSS Mathematical and Statistical System version 9.0 2008 Maple Valley, WA: Aptech Systems Inc

[B17] Saydah SH Loria CM Eberhardt MS Brancati FL Subclinical states of glucose intolerance and risk of death in the U.S Diabetes Care 2001 24 3 447 453 10.2337/diacare.24.3.447 11289466

[B18] Wild S Roglic G Green A Sicree R King H Global prevalence of diabetes: estimates for the year 2000 and projections for 2030 Diabetes Care 2004 27 5 1047 1053 10.2337/diacare.27.5.1047 15111519

[B19] Mainous AG Baker R Koopman J Saxena S Diaz VA Everett CJ Majeed A Impact of the population at risk of diabetes on projections of diabetes burden in the United States: an epidemic on the way Diabetologia 2007 50 5 934 940 10.1007/s00125-006-0528-5 17119914 PMC1849422

[B20] Magliano DJ Barr EL Zimmet PZ Cameron AJ Dunstan DW Colagiuri S Jolley D Owen N Phillips P Tapp RJ Welborn TA Shaw JE Glucose indices, health behaviors, and incidence of diabetes in Australia: the Australian Diabetes, Obesity and Lifestyle Study Diabetes Care 2008 31 2 267 272 10.2337/dc07-0912 17989310

[B21] Gregg EW Gu Q Cheng YJ Narayan KMV Cowie CC Mortality trends in men and women with diabetes, 1971 to 2000 Ann Intern Med 2007 147 3 149 155 17576993 10.7326/0003-4819-147-3-200708070-00167

[B22] Harris MI Klein R Welborn TA Knuiman MW Onset of NIDDM occurs at least 4-7 yr before clinical diagnosis Diabetes Care 1992 15 7 815 819 10.2337/diacare.15.7.815 1516497

[B23] Cowie CC Rust KF Ford ES Eberhardt MS Byrd-Holt DD Li C Williams DE Gregg EW Bainbridge KE Saydah SH Heiss LS Full accounting of diabetes and pre-diabetes in the U.S. population in 1988-1994 and 2005-2006 Diabetes Care 2009 32 2 287 294 10.2337/dc08-1296 19017771 PMC2628695

[B24] Gerstein HC Santaguida P Raina P Morrison KM Balion C Hunt D Yazdi H Booker L Annual incidence and relative risk of diabetes in people with various categories of dysglycemia: a systematic overview and meta-analysis of prospective studies Diabetes Res Clin Pract 2007 78 3 305 312 10.1016/j.diabres.2007.05.004 17601626

[B25] Santaguida PL Balion C Hunt D Morrison K Gerstein H Raina P Booker L Yazdi H Diagnosis, prognosis, and treatment of impaired glucose tolerance and impaired fasting glucose Evid Rep Technol Assess.(Summ.) 2005 128 1 11 16194123 PMC4780988

[B26] Engberg S Vistiesen D Lau C Glumer C Jorgensen T Pedersen O Borch-Johnsen K Progression to impaired glucose regulation and diabetes in the population-based inter99 study Diabetes Care 2009 32 4 606 611 10.2337/dc08-1869 19114617 PMC2660484

[B27] Bonora E Kiechl S Willeit J Oberhollenzer F Egger G Meigs JB Bonadonna RC Muggeo M Population-based incidence rates and risk factors for type 2 diabetes in white individuals: the Bruneck study Diabetes 2004 53 7 1782 1789 10.2337/diabetes.53.7.1782 15220202

[B28] Maskarinec G Erber E Grandinetti A Verheus M Oum R Hopping BN Schmidt MM Uchinda A Juarez DT Hodges K Kolonel LN Diabetes incidence based on linkages with health plans: the multiethnic cohort Diabetes 2009 58 8 1732 1738 10.2337/db08-1685 19258435 PMC2712787

